# Undetectable circulating tumor DNA (ctDNA) levels correlate with favorable outcome in metastatic melanoma patients treated with anti-PD1 therapy

**DOI:** 10.1186/s12967-019-2051-8

**Published:** 2019-09-05

**Authors:** Teofila Seremet, Yanina Jansen, Simon Planken, Hassan Njimi, Mélanie Delaunoy, Hakim El Housni, Gil Awada, Julia Katharina Schwarze, Marleen Keyaerts, Hendrik Everaert, Danielle Lienard, Véronique Del Marmol, Pierre Heimann, Bart Neyns

**Affiliations:** 1 Department of Medical Oncology, Universitair Ziekenhuis Brussel, Vrije Universiteit Brussel (VUB), Laarbeeklaan 101, 1090 Brussels, Belgium; 2 Department of Dermatology, Hôpital Erasme, Université Libre de Bruxelles (ULB), Brussels, Belgium; 3 Department of Biomedical Statistics, Université Libre de Bruxelles (ULB), Brussels, Belgium; 4 Laboratory of Molecular Biology in Haemato-oncology, LHUB-ULB, Hôpital Erasme, Université Libre de Bruxelles (ULB), Brussels, Belgium; 5 Department of Nuclear Medicine, Universitair Ziekenhuis Brussel, Vrije Universiteit Brussel (VUB), Brussels, Belgium

**Keywords:** Translational research, Liquid biopsy, Circulating tumor DNA, Monitoring, Immunotherapy, Metastatic melanoma, BRAF/NRAS mutations monitoring

## Abstract

**Background:**

Treatment with anti-PD1 monoclonal antibodies improves the survival of metastatic melanoma patients but only a subgroup of patients benefits from durable disease control. Predictive biomarkers for durable benefit could improve the clinical management of patients.

**Methods:**

Plasma samples were collected from patients receiving anti-PD1 therapy for ctDNA quantitative assessment of *BRAF*^V600^ and *NRAS*^Q61/G12/G13^ mutations.

**Results:**

After a median follow-up of 84 weeks 457 samples from 85 patients were analyzed. Patients with undetectable ctDNA at baseline had a better PFS (Hazard ratio (HR) = 0.47, median 26 weeks versus 9 weeks, p = 0.01) and OS (HR = 0.37, median not reached versus 21.3 weeks, p = 0.005) than patients with detectable ctDNA. Additionally, the HR for death was lower after the ctDNA level became undetectable during follow-up (adjusted HR: 0.16 (95% CI 0.07–0.36), p-value < 0.001). ctDNA levels > 500 copies/ml at baseline or week 3 were associated with poor clinical outcome. Patients progressive exclusively in the central nervous system (CNS) had undetectable ctDNA at baseline and at subsequent assessments. In multivariate analysis adjusted for LDH, CRP, ECOG and number of metastatic sites, the ctDNA remained significant for PFS and OS. A positive correlation was observed between ctDNA levels and total metabolic tumor volume (TMTV), number of metastatic sites and total tumor burden.

**Conclusions:**

Assessment of ctDNA baseline and during therapy was predictive for tumor response and clinical outcome in metastatic melanoma patients and reflected the tumor burden. ctDNA evaluation provided reliable complementary information during anti-PD1 antibody therapy.

## Background

The treatment of metastatic melanoma has changed significantly during the last decade. Approved medical treatments available are targeted therapy (BRAF and MEK inhibitors) and immunotherapy (immune checkpoint inhibitors—ICI) [1]. Only patients with *BRAF*^V600^—mutant disease (45–50%) can benefit from both targeted and immunotherapy, while all metastatic melanoma patients can receive immunotherapy because predictive biomarkers to select patients for immunotherapy are not available yet in the clinical setting. The first approved immunotherapy showing improved overall survival (OS) for metastatic melanoma patients was ipilimumab, an anti-CTLA-4 blocking monoclonal antibody [2]. The anti-PD-1 monoclonal antibodies, nivolumab and pembrolizumab are the newer generation of ICI providing a higher response rate, longer progression free (PFS) and OS with a lower frequency of adverse events [3, 4]. The ICI enhance the immune system response to melanoma by blocking inhibitory receptors on T lymphocytes. Even though ICI have a significant impact on survival, half of the patients do not respond and will progress on therapy within 1 year. Currently no predictive biomarkers are available to identify patients who benefit most from immunotherapy. Across different tumor types, PD-L1 staining is used to predict response; however melanoma patients with PD-L1 negative tumors can achieve durable objective response [5–7]. Therefore, PD-L1 expression is not used as a predictive biomarker. In this context one technique amply investigated is the analysis of the tumor circulating DNA (ctDNA) present in the plasma of cancer patients [8]. ctDNA has the advantage to be minimally invasive compared to invasive tumor biopsies. Additionally serial blood samples can be easily assessed for successive analyses during therapy to document and potentially predict the course of the disease. Our group and others showed that longitudinal quantitative measurement of *BRAF*^V600mut^ in ctDNA is a useful tool to monitor responses to targeted therapy [9–12]. Furthermore we showed that monitoring during anti-PD1 therapy was informative in a series of seven representative patients with clinical benefit from anti-PD1 treatment [13]. In a prospective trial Lee et al. [14] showed that undetectable ctDNA levels at baseline or within the first 8 weeks is an independent predictor biomarker for response and better OS in a population of patients receiving pembrolizumab or nivolumab ± ipilimumab. Furthermore Cabel et al. [15] showed the same trend in other types of tumors: non-small cell lung cancer, uveal melanoma and microsatellite-instable colorectal cancer. In our prospective translational research study we evaluated metastatic melanoma patients receiving pembrolizumab in monotherapy over a follow up period of almost 2 years with longitudinal *BRAF*^*V600mut*^ and *NRAS*^*Q61/G12/G13mut*^ ctDNA monitoring during treatment.

## Materials and methods

### Patients

The study population was identified among 141 metastatic melanoma patients who participated in an institutional therapeutically non-interventional clinical trial protocol on the outcome of immune-checkpoint inhibition for patients with advanced melanoma at the Universitair Ziekenhuis Brussel (UZ Brussel, Brussels Belgium). Patients were eligible for this sub-study on ctNDA if they had been treated with pembrolizumab and at the condition that *BRAF* V600 or *NRAS* Q61/G12/G13 mutations were detected in tumor tissue and/or plasma samples. Outcome data were collected prospectively between September 2014 and April 2017 in patients who had initiated treatment with the anti-PD1 antibody therapy (pembrolizumab 2 mg/kg 3 weeks) in the early access program prior to reimbursement of the drug in Belgium. Blood samples were prospectively collected after obtaining informed consent with an Ethical Committee (UZ Brussel) approved document. Plasma samples were collected together with routine blood collections (every 2 or 3 weeks during the entire period of immunotherapy).

### ctDNA extraction and PCR analysis by ddPCR and Idylla platform

Silica-based extraction of DNA and subsequent allele-specific quantitative PCR (qPCR) to detect the *BRAF* wild-type gene and the G1798 > A and T1799 > A changes in the *BRAF* gene were performed with Idylla™ (Biocartis) on 1 ml of the stored plasma. The G1798 > A change is present in patients with V600K, V600R, and V600M mutations, whereas the T1799 > A change is present in patients with V600E, V600K, V600E2, and V600D mutations. A linear correlation between Cq values reported by prototype Idylla assays and digital droplet PCR was previously established, allowing precise and sensitive quantification of mutant ctDNA fragments in plasma down to 3 mutant copies per PCR reaction with an analytical sensitivity of 0.01%.

For the detection of *NRAS*^Q61/G12/G13^ mutations DNA was extracted using the QIAamp Circulating Nucleic Acid Kit (Qiagen), followed by droplet digital PCR (ddPCR, Bio-rad). ddPCR was performed using the Bio-Rad QX-200 system (Biorad, Hercules, USA). Assays were purchased from Bio-Rad at 20× concentration (see list below).

**Table:** 

Mutation	Mutation assay	Reference assay
NRAS p.Q61K	dHsaCP2000067	dHsaCP2000068
NRAS p.Q61L	dHsaCP2000069	dHsaCP2000070
NRAS p.Q61R	dHsaCP2000071	dHsaCP2000072
NRAS p.Q61H	dHsaCP2000065	dHsaCP2000066
NRAS p.G12D	dHsaCP2000095	dHsaCP2000096
NRAS P.G13R	dHsaCP2500534	dHsaCP2500535

ddPCR reaction mixtures contained a final concentration of 250 nM for each of the probes, 450 nM for the forward and reverse primers, 1× ddPCR™ Supermix for Probes (No dUTP) (Bio-Rad #186-3024 USA) and 8.8 µl of DNA in a final volume of 22 µl. Twenty microliter of this ddPCR reaction volume were loaded in appropriate wells of a DG8 cartridge (Bio-Rad #186-4008, USA) with 70 µl of generator oil (Bio-Rad #186-3030, USA) into the oil well. Samples are partitioned into approximately 20,000 water–oil emulsion droplets, each 0.85 nl in volume, using the QX200™ Droplet generator™ (Bio-Rad). Forty microliter of this water–oil emulsion were used for the ddPCR assay by transferring it into a 96-wells plate sealed with a PX1™ PCR plate Sealer (Bio-Rad, USA). ddPCR were performed with a T100™ thermal cycler (Bio-Rad, USA) under the following conditions: 1 cycle of 95 °C for 10 min, 40 cycles of 94 °C for 30 s and 55 °C for 1 min, and 1 cycle of 98 °C for 10 min. Cycled droplets were read individually with the QX200TM droplet reader (Bio-Rad). No template control wells (NTC) were included in the assays. Data was analyzed using QuantaSoft TM software version 1.6.6.0320.

### Clinical outcome and statistical analysis

The ctDNA was dichotomized as detectable and undetectable to discriminate between responders and non-responders. Tumor responses were evaluated using the immune-related response criteria in solid tumors (irRC) at 2- or 3-monthly intervals. Patients (n = 7) who had stopped treatment and/or had died before the first response evaluation (all due to clinical progressive disease) were categorized as progressive disease (PD). Patients were grouped in three categories based on their best objective tumor response (BOR): objective response [complete remission (CR) and partial response (PR), CR/PR group], stable disease (SD), and progressive disease (PD). The CR/PR were considered responders, while SD/PD were considered non-responders. PFS and OS were described via the Kaplan–Meier method from the start of therapy to the date of first reported PD or death for PFS and death for OS. Probability of PFS or OS were compared between subgroup using the Log-rank test. In order to determine the adjusted relative risk of death after the undetectable ctDNA occurrence during the follow-up, we developed an extended multivariable Cox model in the overall population and in the defined subgroups. Undetectable ctDNA during the follow-up was introduced in the model as a time-dependent variable using the time to the first occurrence. The nonparametric Mann–Whitney test, one-tailed was used to compare the medians for the ctDNA values between the groups of responders versus non-responders. For additional comparisons of the means between the two groups the unpaired t test with Welch’s correction was used.

In order to identify factors associated with the occurrence of OS and PFS, univariate and multivariable regression analyses were performed using cox regression model. ctDNA, LDH, CRP, number of tumor sites and ECOG variables were considered in the analyses. The values of ctDNA at any time were considered as time-dependent variable. The results are presented as crude and adjusted hazard ratios (HR) with 95% confidence intervals (95% CI). Total metabolic tumor volume (TMTV) was assessed by 18-fluorodeoxyglucose positron emission tomography (18FDG-PET/CT) using MIM Encore SoftwareVR. TMTV was defined as the sum of all tumor-associated voxels with a standardized uptake value (SUV) higher than the mean SUV measured in a reference region in normal liver tissue + 3 standard deviations. Baseline disease burden was determined by the sum of the product of bi-dimensional diameters (SPOD) for every metastasis. Based on the median the patients were divided in three categories: low, intermediate and high burden disease.

Statistical analyses were carried out using Graphpad Prism 7 and IBM^®^ SPSS^®^ statistics software v24. All reported p values are two-sided. The p values lower than 0.05 were considered statistically significant.

## Results

### Patient characteristics and ctDNA analysis

The baseline clinical characteristics of the 85 metastatic melanoma patients with a *BRAF*^V600E/K^ or *NRAS*^Q61/G12/G13^ mutation who were included in the study are summarized in Table 1. The median age was 57 years (range 27–82). The majority of patients had an ECOG of 0 (56 patients [65.9%]) and had stage IV-M1c (AJCC staging 7th edition) at baseline (67 patients [78.8%]). Brain metastases were present in 31 patients (36.5%) and LDH was higher than the upper limit of normal in 37 patients (43.5%).

**Table 1:** Baseline patient characteristics

	ctDNA baseline		All patients (n = 85)	p value
	Undetectable (n = 35)	Detectable (n = 28)		
Median age	59 (35–79)	57.5 (27–82)	57 (27–82)	
Sex				
Female	20 (57.1)	14 (50)	48 (56.5)	0.0572
Male	15 (42.9)	14 (50)	37 (43.5)	
ECOG				
0	29 (82.9)	12 (42.9)	56 (65.9)	0.004
1	4 (11.4)	10 (35.7)	21 (24.7)	
2	2 (5.7)	6 (21.4)	8 (9.4)	
Mutation				
BRAF V600 E/K	24 (68.6)	22 (78.6)	63 (74)	0.374
NRAS Q61K/R/L/H G12D G13R	11 (31.4)	6 (21.4)	22 (26)	
Stage				
III–IV1a, IV1b	11 (31.4)	3 (10.7)	18 (21.2)	0.049
IV-M1c	24 (68.6)	25 (89.3)	67 (78.8)	
Sites ≥ 3				
No	24 (68.6)	7 (25)	38 (44.7)	< 0.001
Yes	10 (28.6)	21 (75)	45 (52.9)	
Unknown	1 (2.9)	0 (0.0)	2 (2.4)	
Brain				
No	21 (60)	21 (75)	54 (63.5)	0.209
Yes	14 (40)	7 (25)	31 (36.5)	
LDH				
≤ ULN	29 (82)	9 (32.1)	46 (54.1)	< 0.001
> ULN	6 (17.1)	18 (64.3)	37 (43.5)	
Unknown	0 (0.0)	1 (3.6)	2 (2.4)	
CRP				
≤ ULN	22 (62.9)	9 (32.1)	38 (44.7)	0.021
> ULN	13 (37.1)	18 (64.3)	45 (52.9)	
Unknown	0 (0.0)	1 (3.6)	2 (2.4)	
BOR				
CR	8 (22.9)	3 (10.7)	13 (15.3)	
PR	3 (8.6)	2 (7.1)	7 (8.2)	
SD	6 (17.1)	3 (10.7)	15 (17.6)	
PD	16 (46)	20 (71.4)	48 (56.5)	
Unknown	2 (5.7)	0 (0.0)	2 (2.4)	
BRAF_MEK treatment				
Yes	20 (57.1)	18 (64.3)	51 (60)	0.693
No	15 (42.9)	10 (35.7)	34 (40)	

Absolute values are shown for each characteristic and percentages are shown between brackets, except for age where the range is shown between brackets. *ULN* upper limit of normal. The p value was calculated using the Fisher exact test/contingency table

**Fig. 1:**
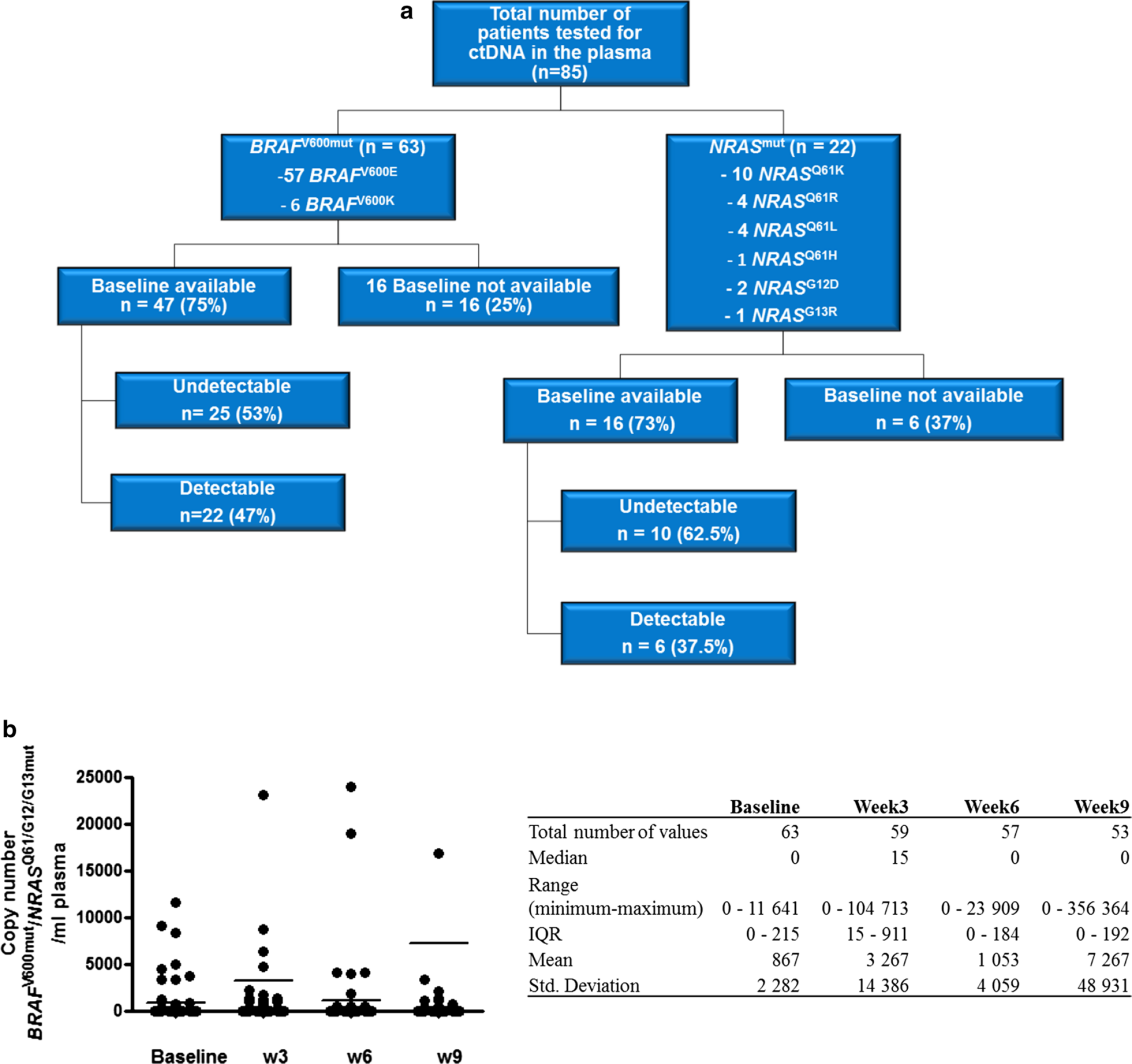
Characterization of samples distribution by type of mutation and baseline ctDNA detection and distribution of absolute values for *BRAF*/*NRAS* mutant copy number. **a** Flowchart of total number of patients analyzed for plasma ctDNA indicating the number of patients presenting a *BRAF* mutation and *NRAS* mutation, the type of mutations and the number of samples available at baseline (pretreatment). Additionally for the baseline samples the number of samples that showed detectable and undetectable ctDNA level is shown for *BRAF* mutations as well as for *NRAS* mutations. **b** Characterization of *BRAF*/*NRAS* mutant copy number distribution for baseline, week 3, 6 and 9 by descriptive statistics (median, range, IQR, mean, std. deviation)

Tumor *BRAF*^V600^ mutations were present in 63 patients (57 *BRAF*^V600E^ and 6 *BRAF*^V600K^) and 22 patients had an *NRAS*^Q61/G12/G13^ mutations (Fig. 1a). These mutations were quantitatively assessed in plasma samples collected every 3 weeks at the occasion of pembrolizumab administration. A total of 495 plasma samples from these 85 patients were analyzed (327 samples for *BRAF*^V600^ mutations and 168 samples for *NRAS*^Q61/G12/G13^ mutations); 63 samples were obtained at baseline (in 35 samples the level of ctDNA was undetectable; 22 samples tested positive for *BRAF*^*V600*^ mutations and 6 samples for *NRAS*^Q61/G12/G13^ mutations). Distribution of ctDNA *BRAF*^*V600*^ and *NRAS*^Q61/G12/G13^ copy numbers (medians and means) for baseline, week 3, 6 and 9 are illustrated in Fig. 1b. In total 457 samples were obtained during treatment (with a median of five samples per patient [range 1 to 19 samples per patient], and a median of five samples for *BRAF*^*V600*^ mutations and seven samples for *NRAS*^Q61/G12/G13^ mutations).

### Correlation between baseline ctDNA analysis and clinical outcome

**Fig. 2:**
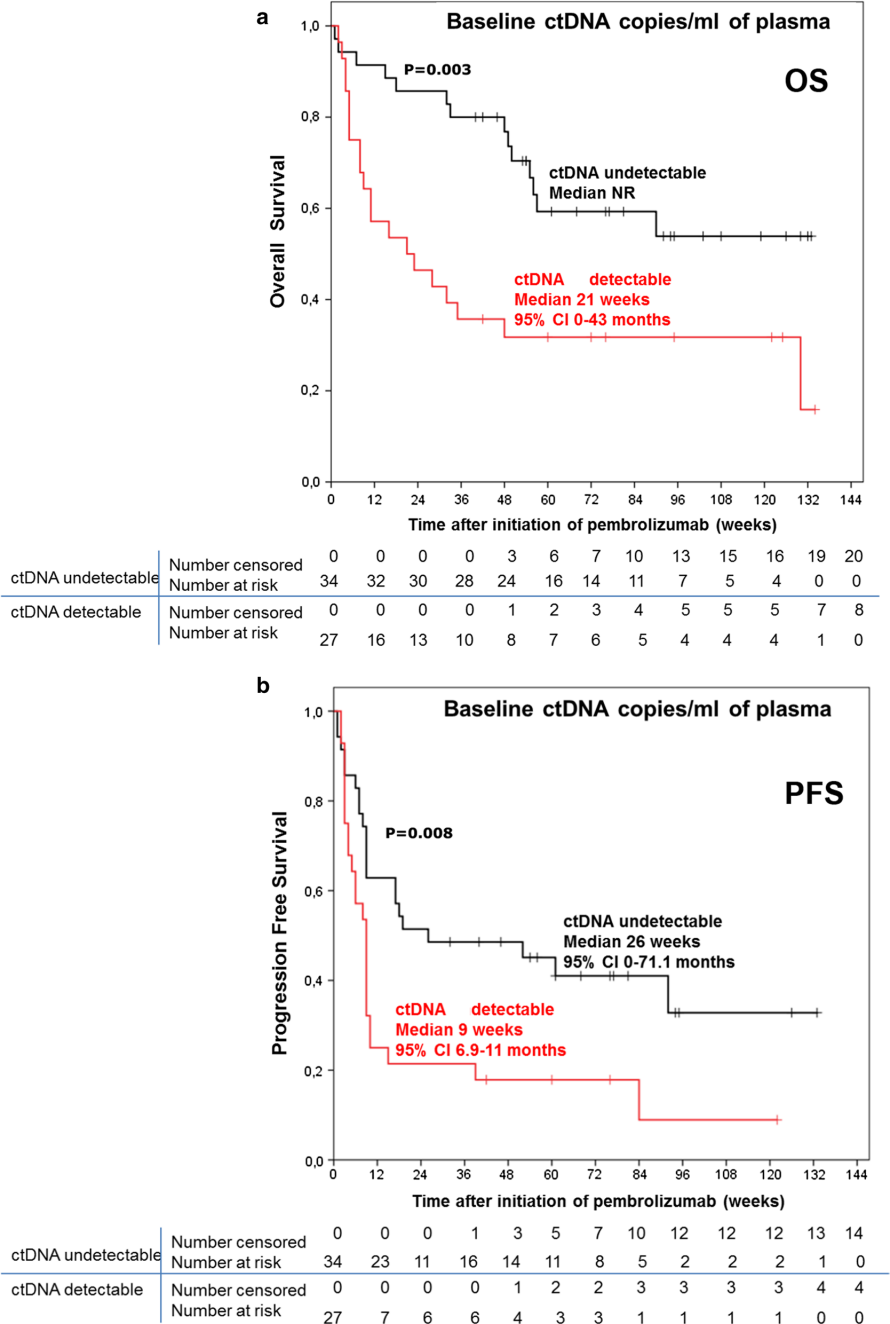
Comparison between the groups of patients with detectable versus undetectable baseline ctDNA for OS and PFS. Kaplan–Meier curve for overall survival (**a**) and progression-free survival (**b**) according to ctDNA levels at baseline (pretreatment)—detectable versus undetectable

**Fig. 3:**
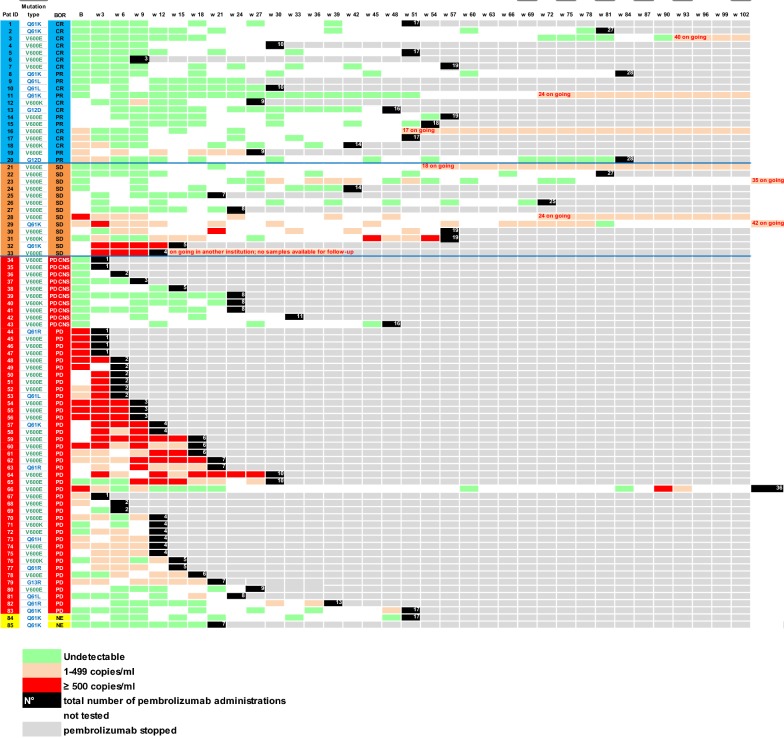
Heatmap showing mutation status and ctDNA longitudinal follow up of individual patients at different points in time during pembrolizumab treatment grouped by BOR [CR, PR, SD, PD exclusively in CNS, PD and not evaluable (NE)] according to irRC. Each row is an individual patient and each column is a time point during follow up (every 3 weeks when pembrolizumab was administrated). The number of pembrolizumab administrations is marked in the black boxes. The grey boxes signify stop of treatment with pembrolizumab. ctDNA levels are marked by green boxes (undetectable), orange boxes (1–499 ctDNA copies per ml of plasma) and red boxes (> 500 ctDNA copies per ml of plasma). Time points at which there was no evaluation of ctDNA are shown in white color

After a median follow-up of 84 weeks (95% CI 63.2–104.8), 60 (70.6%) patients had progressed and 45 (56.2%) had died (2 deaths were not melanoma related). The median PFS and OS of the total study population (n = 85 patients) was 13 weeks (95% CI 9.5–16.5) and 56 weeks (95% CI 7.2–104.8), respectively. The median PFS and OS for the subpopulation of 63 patients who had evaluable plasma samples for baseline ctDNA analysis was 9 weeks (95% CI 4.8–13.1) and 56 weeks (95% CI 7–104.9), respectively. The median OS in the subgroup with undetectable baseline ctDNA (n = 35) was not reached, while in the group of patients with detectable baseline ctDNA the median OS was 21 weeks (95% CI 0–43; p = 0.003) (Fig. 2a). The median PFS in the group with baseline undetectable ctDNA was 26 weeks (95% CI 0–71.1) compared to 9 weeks (95% CI 6.9–11; p = 0.008) for the group with detectable baseline ctDNA (Fig. 2b). The 1-year survival rates were respectively 70% (95% CI 54.3–85.7) in the subgroup with undetectable base line ctDNA levels versus 32% (95% CI 14.36–49.64) in the subgroup with detectable baseline ctDNA levels. Furthermore the 2.5-years survival was 54% (95% CI 34.4–73.6) versus 16% (95% CI − 7.52 to 39.52). Comparing the ctDNA levels in the subgroup of responders (CR/PR) versus non-responders (SD/PD), the median pretreatment ctDNA level in CR/PR-patients was 0 copies/ml of plasma (IQR 0–69) versus 31 copies/ml of plasma (IQR 0–647) for SD/PD patients (Additional file 1: Figure S1A) with a statistically significant difference between the two groups (p = 0.0345, Mann–Whitney test). The baseline ctDNA absolute levels for the two groups (63 patients) are shown in Additional file 1: Figure S1B. The difference for the mean value of *BRAF* and *NRAS* mutant copy number between the two group of patients was statistically significant (p = 0.0052, unpaired t test with Welch’s correction). The mean copy number of *BRAF* and *NRAS* mutant in the responders group (CR/PR) was 42.69 copies (± 19.09) compared to 1199 copies (± 392.7) in the non-responders group. In the CR/PR group the majority of patients had undetectable ctDNA (11 patients—69%), while 5 patients (31%) had detectable ctDNA ranging from 25 to 233 copies/ml of plasma (Fig. 3). In the SD/PD group 22 patients (49%) had undetectable baseline ctDNA and 23 patients (51%) had detectable ctDNA ranging from 31 to 11.641 copies/ml of plasma.

### ctDNA monitoring during the anti-PD1 therapy

**Fig. 4:**
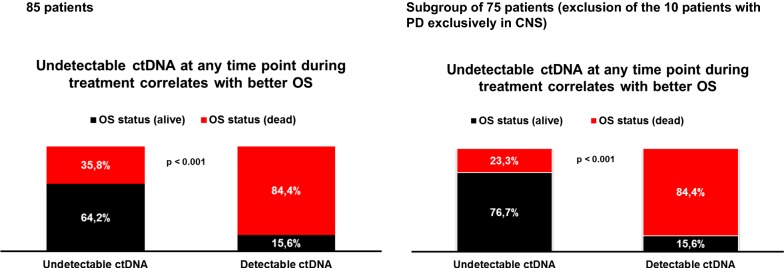
Survival difference between the group of patients who had at least one undetectable ctDNA value during follow up compared with the patients who had ctDNA detectable in the plasma at each time point. Bar diagrams indicating survival status using an extended multivariable Cox model in the overall population (n = 85) and in the defined subgroups (excluding patients with exclusively CNS PD; n = 75) where undetectable ctDNA during the follow-up was introduced in the model as a time-dependent variable using the time to the first occurrence

In an extended multivariable Cox model, undetectable ctDNA during the follow-up was introduced in the model as a time-dependent variable using the time to the first occurrence. The HR for death was lower after the ctDNA level became undetectable during follow-up (adjusted HR: 0.16 (95% CI 0.07–0.36), p-value < 0.001). The difference in OS was statistically significant for the group of patients who had at least one undetectable ctDNA value during follow up compared with the patients who had ctDNA detectable in the plasma at each time point (64.2% alive in the first group versus 15.6% alive in the second group, p < 0.001) (Fig. 4). Moreover in the subpopulation of 75 patients (excluding patients with exclusively brain PD) 76.7% of those who had at least one undetectable ctDNA value during follow up were alive compared to 15.6% alive in the group that had detectable ctDNA in the plasma at each time point (p < 0.001) (Fig. 4). A comparative analysis was performed for *BRAF*/*NRAS* ctDNA levels at second, third and fourth pembrolizumab administration. The median ctDNA at later time points (week 3, 6 and 9) remained 0 (IQR 0–0) in the responders group compared to detectable ctDNA median value in the non-responders group. The difference between the two groups was statistically significant at these three time points (Additional file 2: Figure S2A, D, G). The significant difference in terms of PFS and OS between the patients with undetectable ctDNA and detectable ctDNA was observed as well at these subsequent three time points: at week 3 median PFS was 89 weeks (95% CI 20.5–157.4) versus 6 weeks (95% CI 4.9–7.1) (p < 0.001) and median OS not reached versus 25 weeks (95% CI 9.2–40.7) (p < 0.001); at week 6 median PFS was estimated at 86 weeks (95% CI not available) versus 3 weeks (95% CI 2.3–3.6) (p < 0.001) and median OS not reached versus 42 weeks (95% CI 15.2–68.7) (p = 0.002); at week 9 median PFS was estimated at 83 weeks (95% CI not available; 75th percentiles 9) versus 0 (95% CI not available; 75th percentiles 0, at the median all patients had PD) weeks (p = 0.001) and median OS not reached (75th percentiles 81 weeks) versus 26 weeks (95% CI 0.7–51.2) (p = 0.002). The Kaplan–Meier curves are shown in Additional file 2: Figure S2B, C, E, F, H, I.

### ctDNA levels in the subgroup of patients progressive exclusively in the CNS

Within the subgroup of “PD patients”, we identified 10 patients who presented with PD exclusively in the central nervous system (CNS). The majority of the patients (9/10) had CNS involvement when starting anti-PD-1 therapy and one patient developed brain metastases during treatment (MEL40). These patients had undetectable ctDNA at baseline and at subsequent assessments (36 samples analyzed in total). In this subgroup 9 patients had *BRAF*^V600E^ mutations and 1 patient had a *BRAF*^V600K^ mutation. The median number of samples tested per patient was 3 (ranging from 1 to 8 samples per patient) with a median of 7 pembrolizumab administrations (ranging from 1 to 16). All samples showed undetectable ctDNA even at later time points after week 9 (Additional file 3: Table S1). The median PFS in this subgroup was 3 weeks (95% CI 0–6.8) and the median OS was 33 weeks (95% CI 0–85.7) (Additional file 4: Figure S3A). An interesting finding was observed for patient MEL36 that showed PD with multiple brain metastases under targeted therapy (dabrafenib 150 mg bid/trametinib 2 mg qd) and received pembrolizumab as second line treatment. This patient was presenting a PR in the visceral metastases under targeted therapy with no metabolic activity on the PET-CT. The analysis of the plasma ctDNA at baseline showed undetectable levels despite the PD in the brain. Early on during pembrolizumab treatment the ctDNA levels became detectable in parallel with PD observed as well in the visceral metastases in addition to the brain PD (Additional file 4: Figure S3B).

### High baseline or on-treatment levels of *BRAF*/*NRAS* mutations in ctDNA correlate with poor clinical outcome

In the PD group 11 out of 50 patients (22%) had baseline ctDNA levels higher than 500 copies/ml. For this subgroup the median PFS was 5 weeks (95% CI 3–6.9) and median OS was 8 weeks (95% CI 4.8–11.1) (Additional file 4: Figure S3C). Eight additional patients in the PD group had more than 500 copies/ml at week 3 assessment (no baseline available for 6 patients and 2 patients had increased ctDNA levels from baseline to week 3: 31 to 1294 copies/ml and 177 to 986 copies/ml) (Additional file 3: Table S2). For this additional subgroup of 8 patients, the median PFS was 6 weeks (95% CI 3.2–8.7) and the median OS was 11 weeks (95% CI 5.5–16.5) (Additional file 4: Figure S3D). The median value for ctDNA at baseline in the group of 11 patients was 3 652 (IQR 1241–8322) copies/ml and for the group of 8 patients was 1 973 (IQR 1140–27,192) copies/ml. In these two groups the majority of the patients (17/19; 89%) had baseline LDH higher than the upper limit of normal, and baseline CRP was higher than the upper limit of normal in 14/19 (74%) patients. Additionally, the majority [14/18 (78%); 1 was NE] had more than three metastatic sites. Moreover, an important increase in the ctDNA levels was observed (from 104.5 copies at baseline to 1980 copies/ml only 4 days after the first pembrolizumab administration) in one patient (MEL 67) who died 3 weeks later (data not shown). This patient had normal baseline LDH, elevated CRP (> 10xN) and more than three metastatic sites.

### Multivariable analysis

The multivariable Cox regression analysis including LDH, CRP, number of metastatic sites (> 3) and ECOG variables showed that undetectable ctDNA at baseline remained significantly correlated with PFS and OS (Table 2). Additionally, the ECOG status and baseline CRP remained significant for PFS, while for OS only the number of metastatic sites remained an additional significant co-variate. This analysis was performed in the subpopulation of 75 patients (excluding CNS exclusively PD patients).

**Table 2:** Multivariable analysis

Variable	PFS as the dependent variable				OS as the dependent variable			
	Univariate		Multivariable		Univariate		Multivariable	
	HR (95% CI)	p-value	HR (95% CI)	p-value	HR (95% CI)	p-value	HR (95% CI)	p-value
Baseline LDH	2.79 (1.55–5.03)	< 0.001			3.37 (1.65–6.89)	< 0.001		
Baseline CRP	2.38 (1.28–4.42)	0.006	2.16 (1.15–4.07)	0.017	2.43 (1.17–5.06)	0.017		
ECOG	2.87 (1.20–6.90)	0.018	2.67 (1.04–6.84)	0.04	4.02 (1.65–9.79)	0.002		
Number of metastatic sites	2.33 (1.28–4.26)	0.006			3.06 (1.44–6.52)	0.004	2.38 (1.10–5.14)	0.027
Undetectable ctDNA	0.18 (0.09–0.35)	< 0.001	0.20 (0.10–0.40)	< 0.001	0.13 (0.06–0.29)	< 0.001	0.16 (0.07–0.36)	< 0.001

Univariate and multivariable regression analyses were performed using cox regression model. Factors associated with the occurrence of OS and PFS (ctDNA, LDH, CRP, number of tumor sites and ECOG variables) were considered in the analyses. The results are presented as hazard ratios with 95% confidence intervals and p values

### Correlation between baseline ctDNA levels and disease burden

**Fig. 5:**
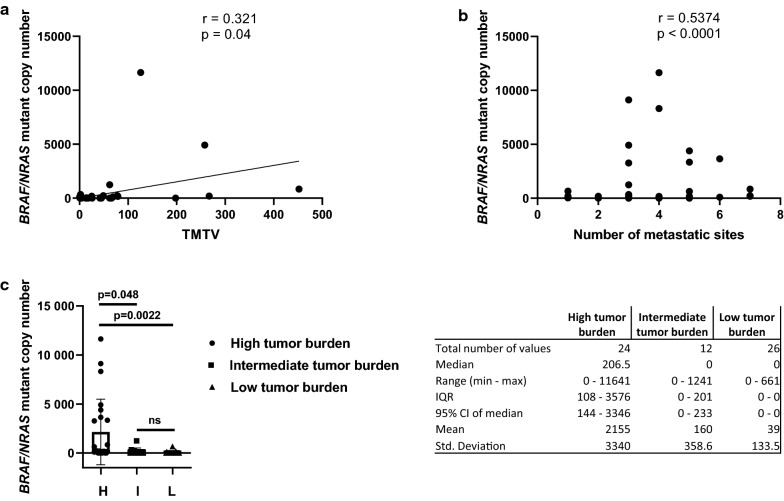
Tumor burden and ctDNA correlation. Scatterplot of *BRAF/NRAS* mutant copy number (y-axis) and total metabolic tumor volume (x-axis) (**a**). Scatterplot of *BRAF/NRAS* mutant copy number (y-axis) distribution in terms of number of metastatic sites (x-axis) (**b**). Box plots detailing the ctDNA median copy numbers for the three groups of patients: high tumor burden (H), intermediate tumor burden (I) and low tumor burden (L) at baseline evaluation (**c**). Characterization of *BRAF*/*NRAS* mutant copy number distribution for baseline in the three groups by descriptive statistics (median, range, IQR, mean, std. deviation)

Baseline ctDNA levels correlated with total metabolic tumor volume (TMTV) assessed by 18FDG-PET/CT (Fig. 5a). We found a positive correlation between ctDNA levels and TMTV (Pearson correlation r = 0.321, p = 0.04). The TMTV evaluation was available in 40 patients. Additionally the total tumor burden (volume of the metastatic disease) and the number of metastatic sites were assessed. This assessment was available for 62 patients. A positive correlation was observed between the number of metastatic sites and ctDNA levels (Spearman correlation, r = 0.5374, p < 0.0001) (Fig. 5b). The patients were divided in three groups after tumor burden assessment: low, intermediate and high tumor burden. The comparison between the group showed a significant difference between the low and high tumor burden group (p = 0.0022; median 206.5 [95% CI 144–3346] versus 0 [95% CI 0–0]) and between the high and intermediate tumor burden group (p = 0.048; median 206.5 [95% CI 144–3346] versus 0 [95% CI 0–233]) (Fig. 5c).

## Discussion

PD-1 immune checkpoint inhibitors have become a standard of care for patients diagnosed with metastatic melanoma. However, since the majority of patients remains in need of alternative treatment options there is a high need for the identification of reliable novel biomarkers that can predict response and probability for long-term PFS, detect early disease progression, and assist in timely clinical decision making. The recent research is focused on the characterization of tumor immune microenvironment together with the analysis of mutational tumor burden. Current findings showed that a T cell inflamed gene expression and the mutational burden of the tumor are independent prognostic factors for the response to anti-PD1 therapy across tumor types [16]. Additionally the MHC I expression predicted resistance to anti-CTLA4 therapy, while MHC class II membrane expression and interferon-γ (IFN-γ) gene signatures predicted response to anti-PD-1 [17]. Moreover although the PD-L1 expression does not predict response in all patients, it can give an information regarding a higher probability of response but being only one factor among many that affect how patients respond [18, 19]. Another additional promising candidate biomarkers is the use of the real-time ctDNA analysis [20]. A growing number of findings are available in different type of cancers regarding the ability of ctDNA to be informative for response to therapies, relapse detection or resistance mechanism uncovering [21, 22]. The first ctDNA liquid biopsy approved for use in clinical settings was in lung cancer patients for the identification of *EGFR* mutations for first-line therapy or identifying resistance mutations that will allow for treatment with third generation EGFR inhibitors [23, 24]. In melanoma there is no liquid biopsy test approved in the clinical settings at present. Nevertheless several studies showed the utility of ctDNA as a diagnostic, predictive and prognostic biomarker [9–11, 13, 14]. Thus the landscape for ctDNA utilization in clinical setting may change in the near future for melanoma patients especially for diagnostic purposes in advanced metastatic emergency situations if tumor biopsy is invasive and risky or the tissue results are not informative or will be available after a significant delay. Moreover, ctDNA can also ease the decision in the daily clinical practice when radiological evaluation is problematic especially for patients receiving immunotherapy. In this context, an important advantage of ctDNA is the possibility of non-invasive serial testing for monitoring treatment response and resistance to therapy. Regarding the predictive value for ctDNA in melanoma there is one study showing that ctDNA level is an independent predictive biomarker for response, PFS and OS in melanoma patients receiving anti-PD1 antibody-based therapy [14]. Our results confirm these observations. In their study the patients received pembrolizumab or nivolumab ± ipilimumab, while our study population was more homogeneous all patients receiving pembrolizumab monotherapy. In our population similar findings were observed: undetectable baseline or early decline to undetectable ctDNA levels (during the first 6–9 weeks) correlated with prolonged PFS and OS. This pattern was observed in the CR/PR group. An additional study showed ctDNA correlation with response and PFS but again in a heterogeneous population where patients received either targeted therapy or immunotherapy [25]. In line with previous observations in melanoma patients [14, 25], the subgroup of patients among the PD patients that had exclusive PD in the CNS presented undetectable ctDNA levels at any time point during treatment. This was also observed in breast and lung cancer patients with restricted CNS disease thus it seems to be a general observation [26–29]. One hypothesis is that the blood–brain barrier plays a major role for this event because ctDNA can be easily detected in cerebrospinal fluid (CSF) of patients with brain lesions. Moreover, it can be the modality of choice to confirm metastatic disease in patients with leptomeninges carcinomatosis or brain lesions with limited accessibility or difficult to sample lesions due to the high invasiveness of the biopsy procedure.

Additionally, high ctDNA levels (> 500 copies/ml) at baseline or week 3 observed only in the group of PD patients were indicative of a very poor clinical outcome. Moreover, in the PD group the patients with ctDNA levels < 500 copies/ml at baseline never presented a decline to undetectable ctDNA during the first 9 weeks. A cut-off for OS lower or higher than 2 years for patients on targeted therapy was shown to be 216 mutated copies/ml in the manuscript of Sanmamed et al. [12]. Finding a precise cut-off informative for response to therapy is still a challenge at this point in time. We hypothesize that high ctDNA corresponds mainly to high proliferative activity/status of tumor cells (aggressive tumor phenotype with poor clinical outcome), which in the majority of the patients correlates as well with high tumor burden.

An added value for our approach is that using the cartridge system on Idylla platform for mutation analysis, the results can be available the same day as plasma collection rendering possible a clinical decision taking also into account ctDNA level. This can be extremely important when the interpretation of the radiological evaluation is problematic for patients receiving immunotherapy. With the development of cartridges testing multiple genes and implicitly multiple mutations, this technique can be used to identify, besides the dynamics of the disease, the mutational spectrum from disease sites. However, advancements are needed to overcome the difficulties related to undetectable ctDNA levels when progression occurs in cerebral sites. This should be considered if the technique will be introduced as routine clinical practice. This observation reinforces the importance of brain MRI or CT use during follow up of metastatic melanoma patients. Nevertheless, for the majority of the patients ctDNA monitoring was informative for tumor response or disease progression.

## Conclusion

In clinics, ctDNA provides reliable complementary information to imaging during anti-PD-1 antibody therapy improving the clinical follow-up and evaluation of the efficacy of anti-PD-1 therapy. ctDNA assessment was predictive for tumor response and clinical outcome in metastatic melanoma patients except for patients with disease progression exclusively in the CNS. An added value for our approach is that using an automatized cartridge system, the results can be available the same day as plasma collection rendering possible in the future a clinical decision taking into account the ctDNA level.

## Supplementary information

**Additional file 1: Figure S1.** Median and mean for *BRAF*/*NRAS* mutant copy number for responders (CR/PR) versus non-responders (SD/PD). Box plots showing the median (C) and mean (D) baseline ctDNA copy numbers compared in objective responders (CR/PR) and non-responders (SD/PD).

**Additional file 2: Figure S2.** Comparison between the groups of patients with detectable versus undetectable ctDNA for OS/PFS and *BRAF*/*NRAS* mutant median copy number for responders (CR/PR) versus non-responders (SD/PD) during follow up at week 3, 6 and 9. Box plots detailing the ctDNA median copy numbers for responders (CR/PR) versus non-responders (SD/PD) at the second pembrolizumab cycle in week 3 (A), the third pembrolizumab cycle in week 6 (D) and the fourth pembrolizumab cycle in week 9 (G). Kaplan-Meyer curves comparing PFS (B, E, H) and OS (C, F, I) in patients with detectable or undetectable ctDNA levels at that time point, respectively at weeks 3, 6 and 9.

**Additional file 3: Table** **S1.** Longitudinal monitoring of patients with progressive disease exclusively in the central nervous system. **Table** **S2.** Absolute values of longitudinal monitoring of patients with > 500 copies of mutated ctDNA/ml of plasma at baseline and/or at week 3.

**Additional file 4: Figure** **S3.** Kaplan–Meier survival curves for OS and PFS for patients with exclusively CNS PD and patients with *BRAF*/*NRAS* mutant copy number of > 500 copies/mL of plasma. (A) OS and PFS for the subgroup with PD exclusively in the CNS (n = 10), time is shown on the horizontal axis in weeks; (B) For Patient MEL36 the *BRAF*^V600E/D^ mutation copy number is shown on the left vertical axis and the fractional abundance (% from the total cell-free DNA) on the right vertical axis. Time (in days) is shown on the horizontal axis. Illustrative CT or PET/CT images are shown on the upper part on the chart. The ctDNA was undetectable pretreatment with pembrolizumab when PD brain was observed on brain MRI and became detectable when the disease progressed as well in the visceral metastatic sites. (C) OS and PFS (in weeks) for the subgroup of patients with a baseline ctDNA copy number of > 500 copies/mL of plasma (n = 11) and (D) the subgroup of patients with a copy number of > 500 copies/mL of plasma at week 3 (n = 8).

## Data Availability

All raw data are available for review if necessary.

## Abbreviations

Anti-PD1: anti-programmed cell death 1
DNA: deoxyribonucleic acid
*BRAF*: B-Raf proto-oncogene, serine/threonine kinase
*NRAS*: NRAS proto-oncogene, GTPase
ctDNA: circulating tumor DNA
ddPCR: Droplet Digital polymerase chain reaction
PFS: progression free survival
(a)HR: (adjusted) hazard ratio
PD: progressive disease
CNS: central nervous system
LDH: lactate dehydrogenase
CRP: C-reactive protein
ECOG: Eastern Cooperative Oncology Group
MEK: mitogen-activated protein kinase kinase 1
ICI: immune checkpoint inhibition
OS: overall survival
CTLA-4: cytotoxic T-lymphocyte associated protein 4
PD-L1: programmed cell death 1 ligand 1
qPCR: quantitative polymerase chain reaction
CI: confidence interval
EAP: Expanded Access Program
AJCC: American Joint Committee on Cancer
BOR: best overall response
CR: complete response
PR: partial response
SD: stable disease
PET/CT: positron emission tomography/computed tomography
IQR: interquartile range
NE: not evaluated
N: normal
*EGFR*: epidermal growth factor receptor
CSF: cerebrospinal fluid
MRI: magnetic resonance imaging
bid: twice daily
qd: once daily
