# Supplementary figures and images for: Undetectable circulating tumor DNA (ctDNA) levels correlate with favorable outcome in metastatic melanoma patients treated with anti-PD1 therapy
Source: J Transl Med. 2019 Sep 5;17:303. doi: 10.1186/s12967-019-2051-8 (PMC6727487; doi:10.1186/s12967-019-2051-8)

Additional file 1: Figure S1.

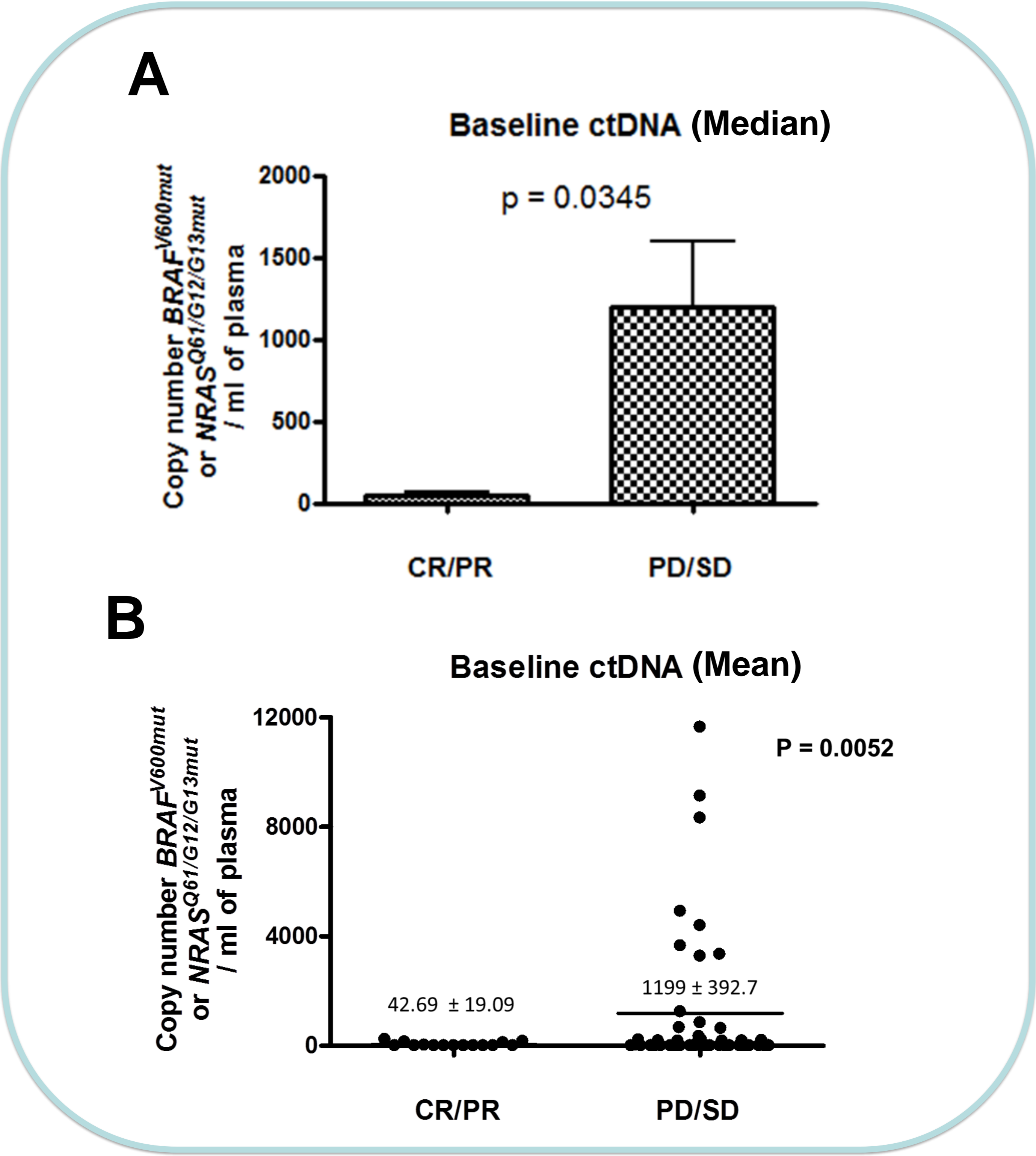

Supplement: Supplementary file 1 — Additional file 1: Figure S1. Median and mean for BRAF/NRAS mutant copy number for responders (CR/PR) versus non-responders (SD/PD). Box plots showing the median (C) and mean (D) baseline ctDNA copy numbers compared in objective responders (CR/PR) and non-responders (SD/PD). [file 12967_2019_2051_MOESM1_ESM.pdf]

Additional file 2: Figure S2

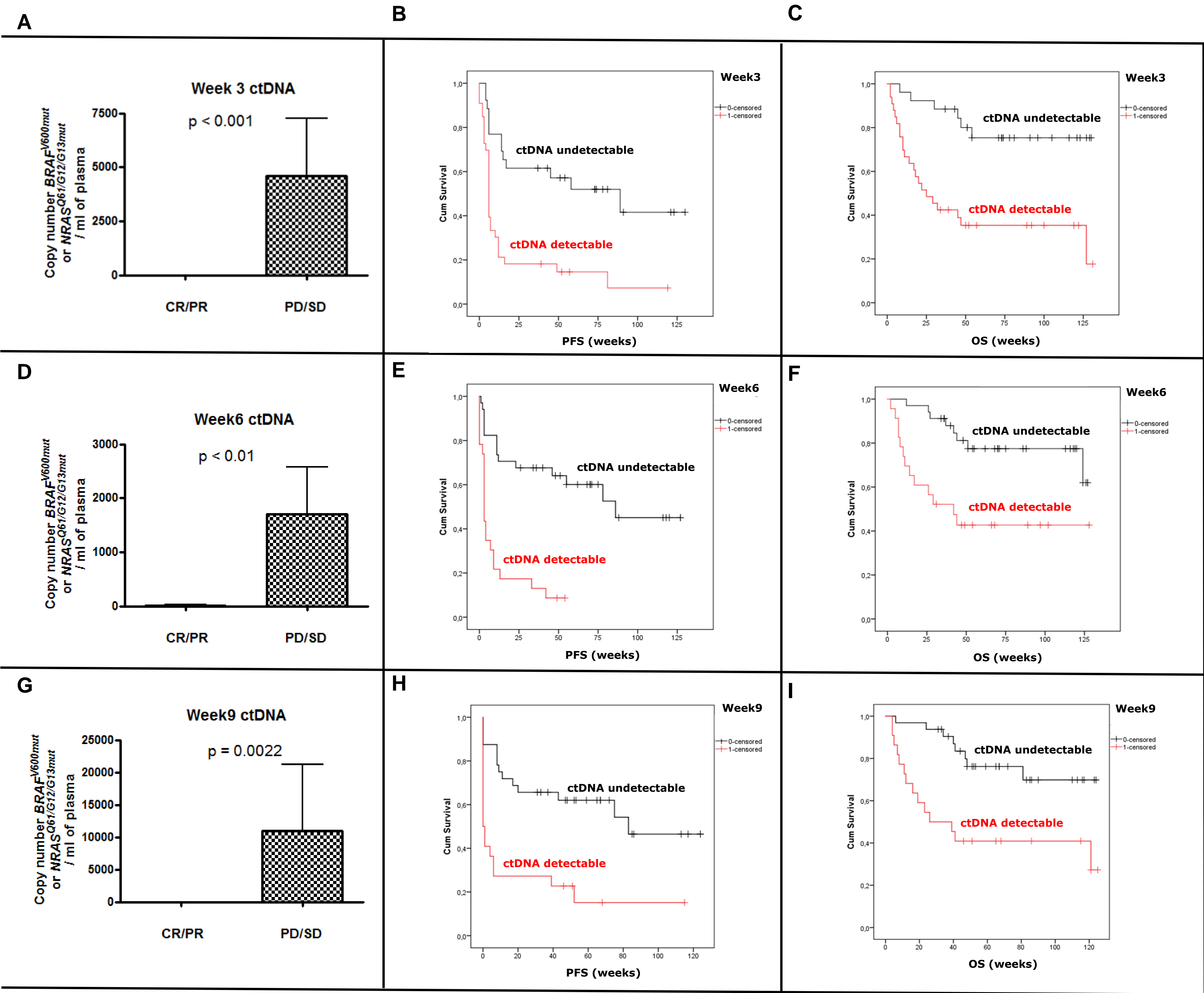

Supplement: Supplementary file 2 — Additional file 2: Figure S2. Comparison between the groups of patients with detectable versus undetectable ctDNA for OS/PFS and BRAF/NRAS mutant median copy number for responders (CR/PR) versus non-responders (SD/PD) during follow up at week 3, 6 and 9. Box plots detailing the ctDNA median copy numbers for responders (CR/PR) versus non-responders (SD/PD) at the second pembrolizumab cycle in week 3 (A), the third pembrolizumab cycle in week 6 (D) and the fourth pembrolizumab cycle in week 9 (G). Kaplan-Meyer curves comparing PFS (B, E, H) and OS (C, F, I) in patients with detectable or undetectable ctDNA levels at that time point, respectively at weeks 3, 6 and 9. [file 12967_2019_2051_MOESM2_ESM.pdf]
