# Supplementary material for: Undetectable circulating tumor DNA (ctDNA) levels correlate with favorable outcome in metastatic melanoma patients treated with anti-PD1 therapy
Source: J Transl Med. 2019 Sep 5;17:303. doi: 10.1186/s12967-019-2051-8 (PMC6727487; doi:10.1186/s12967-019-2051-8)
Supplement: Supplementary file 3 — Additional file 3: Table S1. Longitudinal monitoring of patients with progressive disease exclusively in the central nervous system. Table S2. Absolute values of longitudinal monitoring of patients with > 500 copies of mutated ctDNA/ml of plasma at baseline and/or at week 3. [file 12967_2019_2051_MOESM3_ESM.docx]

**Additional file 3: Table** **S1. Monitoring of patients with progressive disease exclusively in the central nervous system.**

| Patient ID | Mutation Type | N°of treatment cycles | Copy Number *BRAF* V600E/K mut/ml of plasma | | | | | | | | | | |
| --- | --- | --- | --- | --- | --- | --- | --- | --- | --- | --- | --- | --- | --- |
|  |  |  | **baseline** | **W3** | **W6** | **W9** | **W12** | **W15** | **W18** | **W21** | **W24** | **W27** | **W45** |
| MEL34 | **BRAF V600E** | **1** | **0** |  |  |  |  |  |  |  |  |  |  |
| MEL40 | **BRAF V600K** | **8** | **0** |  |  | **0** | **0** | **0** |  | **0** |  |  |  |
| MEL35 | **BRAF V600E** | **1** | **0** |  |  |  |  |  |  |  |  |  |  |
| MEL43 | **BRAF V600E** | **16** | **0** |  |  |  | **0** |  |  |  |  | **0** | **0** |
| MEL41 | **BRAF V600E** | **8** | **0** | **0** | **0** | **0** |  | **0** | **0** |  |  |  |  |
| MEL42 | **BRAF V600E** | **11** | **0** |  |  | **0** |  |  | **0** |  |  |  |  |
| MEL38 | **BRAF V600E** | **5** | **0** |  |  |  | **0** |  |  |  |  |  |  |
| MEL37 | **BRAF V600E** | **3** | **0** | **0** | **0** |  |  |  |  |  |  |  |  |
| MEL39 | **BRAF V600E** | **8** | **0** | **0** | **0** | **0** | **0** | **0** | **0** | **0** |  |  |  |
| MEL36 | **BRAF V600E** | **2** | **0** |  |  |  |  |  |  |  |  |  |  |

Each row represents an individual patient, the type of mutation and number of pembrolizumab administrations are shown in the second and third column.

**Additional file 3: Table** **S2. Monitoring of patients with > 500 copies of mutated ctDNA/ml of plasma at baseline and/or at week 3 (absolute values).**

| Patient ID | Mutation Type | N°of treatment cycles | Copy Number *BRAF* V600E/K mut/ml of plasma | | | | | | | | | |
| --- | --- | --- | --- | --- | --- | --- | --- | --- | --- | --- | --- | --- |
|  |  |  | **baseline** | **W3** | **W6** | **W9** | **W12** | **W15** | **W18** | **W21** | **W24** | **W27** |
| MEL44 | **NRAS**  **Q61R** | **1** | **841** |  |  |  |  |  |  |  |  |  |
| MEL45 | **BRAF V600E** | **1** | **3272** |  |  |  |  |  |  |  |  |  |
| MEL46 | **BRAF V600E** | **1** | **8322** |  |  |  |  |  |  |  |  |  |
| MEL47 | **BRAF V600E** | **1** | **9120** |  |  |  |  |  |  |  |  |  |
| MEL48 | **BRAF V600E** | **2** | **3346** | **4661** |  |  |  |  |  |  |  |  |
| MEL49 | **BRAF V600E** | **2** | **4397** |  |  |  |  |  |  |  |  |  |
| MEL50 | **BRAF V600E** | **2** |  | **104713** |  |  |  |  |  |  |  |  |
| MEL51 | **BRAF V600E** | **2** |  | **23110** |  |  |  |  |  |  |  |  |
| MEL52 | **BRAF V600E** | **2** | **31** | **1294** |  |  |  |  |  |  |  |  |
| MEL53 | **NRAS Q61L** | **2** | **177** | **986** |  |  |  |  |  |  |  |  |
| MEL54 | **BRAF V600E** | **3** | **3652** | **6324** | **4126** |  |  |  |  |  |  |  |
| MEL55 | **BRAF V600E** | **3** | **4913** | **8749** | **4012** |  |  |  |  |  |  |  |
| MEL56 | **BRAF V600E** | **3** | **11641** | **1078** | **4126** |  |  |  |  |  |  |  |
| MEL57 | **NRAS Q61K** | **4** |  | **31273** | **23909** | **356364** |  |  |  |  |  |  |
| MEL58 | **BRAF V600E** | **4** |  | **939** | **195** | **3394** |  |  |  |  |  |  |
| MEL59 | **BRAF V600E** | **6** |  | **1756** | **18967** | **16788** | **1652** | **11641** |  |  |  |  |
| MEL60 | **BRAF V600E** | **6** | **633** | **911** | **98** | **1049** | **282** | **98** |  |  |  |  |
| MEL64 | **BRAF V600E** | **10** |  | **2189** | **234** |  | **712** | **120** | **897** | **747** | **703** | **703** |
| MEL66 | **BRAF V600E** | **36** | **1241** | **102** | **0** | **78** | **0** | **0** | **0** | **0** |  |  |

Each row represents an individual patient, the type of mutation and number of pembrolizumab administrations are shown in the second and third column.

**Legends for Tables**

**Additional file 3: Table** **S1. Longitudinal monitoring of patients with progressive disease exclusively in the central nervous system**

Each row represents an individual patient, the type of mutation and number of pembrolizumab administrations are shown in the second and third column.

*Patient MEL36 had massive PD in the CNS (nodular lesions meningeal hemi cerebellar right, punctiform lesions spread in the cerebellum, punctiform lesions in the basal ganglia, left frontal cortex, central sulcus right and disseminated pineal leptomeningeal metastatses) with regression of all visceral metastases on BRAF/MEK inhibition. At baseline the ctDNA was undetectable and later at 5 days before the second administration of pembrolizumab the value for ctDNA (BRAF V600E) was 7 copies/ml of plasma followed by a value of 22 copies/ml at 5 days after the second pembrolizumab administration. At this time point the PET/CT evaluation showed additional PD in the visceral metastases.

**Additional file 3: Table** **S2. Absolute values of longitudinal monitoring of patients with > 500 copies of mutated ctDNA/ml of plasma at baseline and/or at week 3**

Each row represents an individual patient, the type of mutation and number of pembrolizumab administrations are shown in the second and third column.

**Legends for Figures**

**Additional file 1: Figure S1.** **Median and mean for *BRAF*/*NRAS* mutant copy number for responders (CR/PR) versus non-responders (SD/PD).** Box plots showing the median (C) and mean (D) baseline ctDNA copy numbers compared in objective responders (CR/PR) and non-responders (SD/PD).

**Additional file 2: Figure S2.** **Comparison between the groups of patients with detectable versus undetectable ctDNA for OS/PFS and *BRAF*/*NRAS* mutant median copy number for responders (CR/PR) versus non-responders (SD/PD) during follow up at week3, 6 and 9.** Box plots detailing the ctDNA median copy numbers for responders (CR/PR) versus non-responders (SD/PD) at the second pembrolizumab cycle in week 3 (A), the third pembrolizumab cycle in week 6 (D) and the fourth pembrolizumab cycle in week 9 (G). Kaplan-Meyer curves comparing PFS (B, E, H) and OS (C, F, I) in patients with detectable or undetectable ctDNA levels at that time point, respectively at weeks 3, 6 and 9.

**Additional file 4: Figure S3.** **Kaplan-Meier survival curves for OS and PFS for patients with exclusively CNS PD and patients with *BRAF*/*NRAS* mutant copy number of >500 copies/mL of plasma.** (A) OS and PFS for the subgroup with PD exclusively in the CNS (n=10), time is shown on the horizontal axis in weeks; (B) For Patient MEL36 the *BRAF* ^V600E/D^ mutation copy number is shown on the left vertical axis and the fractional abundance (% from the total cell-free DNA) on the right vertical axis. Time (in days) is shown on the horizontal axis. Illustrative CT or PET/CT images are shown on the upper part on the chart. The ctDNA was undetectable pretreatment with pembrolizumab when PD brain was observed on brain MRI and became detectable when the disease progressed as well in the visceral metastatic sites. (C) OS and PFS (in weeks) for the subgroup of patients with a baseline ctDNA copy number of >500 copies/mL of plasma (n=11) and (D) the subgroup of patients with a copy number of >500 copies/mL of plasma at week 3 (n=8).
