# Supplementary material for: Undetectable circulating tumor DNA (ctDNA) levels correlate with favorable outcome in metastatic melanoma patients treated with anti-PD1 therapy
Source: J Transl Med. 2019 Sep 5;17:303. doi: 10.1186/s12967-019-2051-8 (PMC6727487; doi:10.1186/s12967-019-2051-8)

Additional file 4: Figure S3

A. Subgroup of 10 patients with PD exclusively in CNS

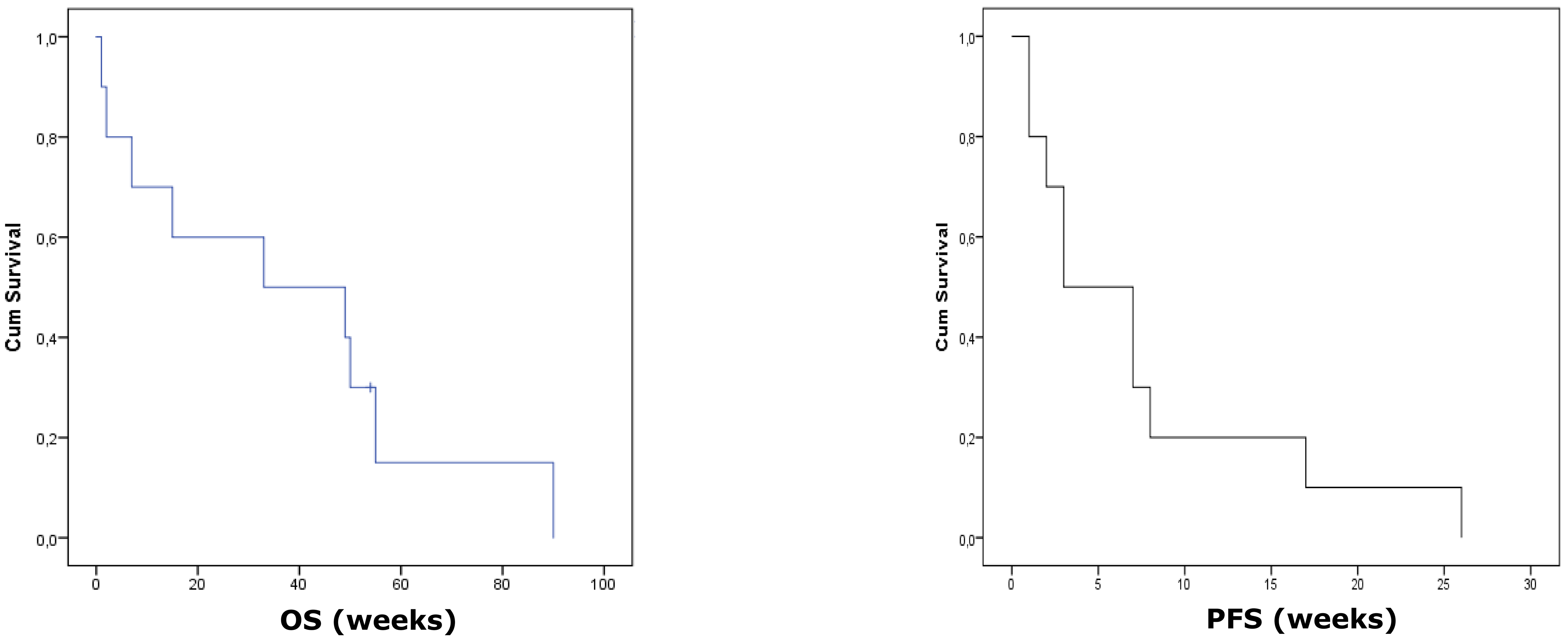

B. Patient MEL36 monitoring by plasma ctDNA and representative radiological evaluation of the disease course

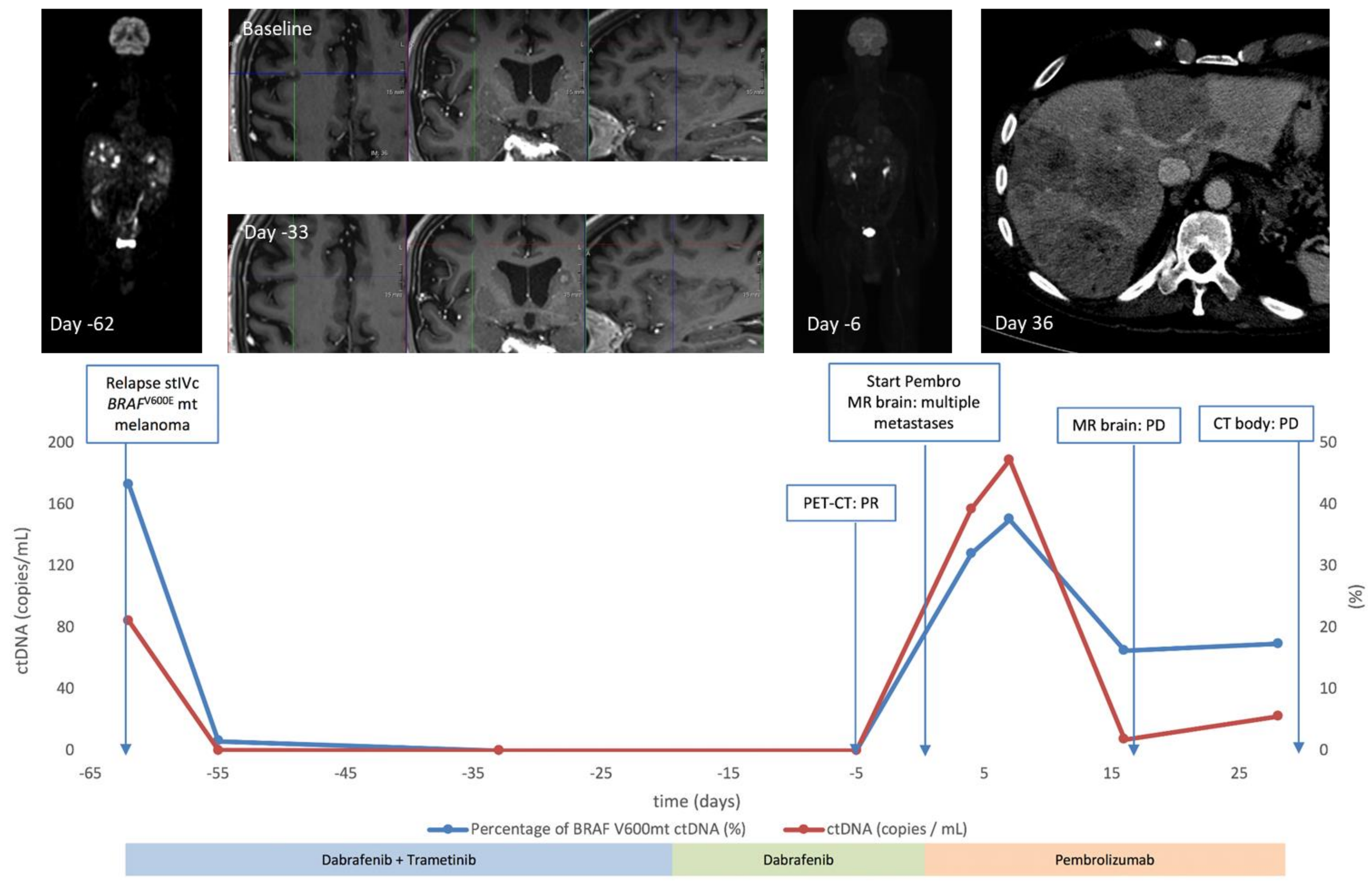

C. Subgroup of 11 patients with baseline ctDNA > 500 copies/ml of plasma

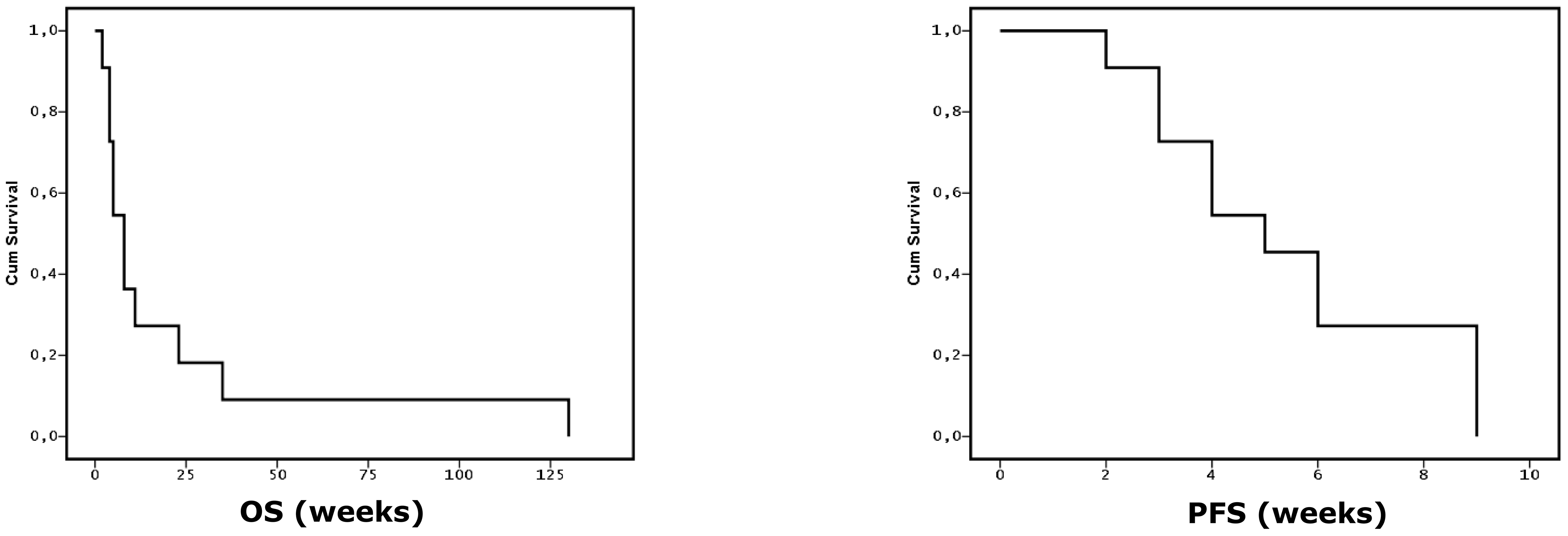

D. Subgroup of 8 patients with w3 ctDNA > 500 copies/ml of plasma

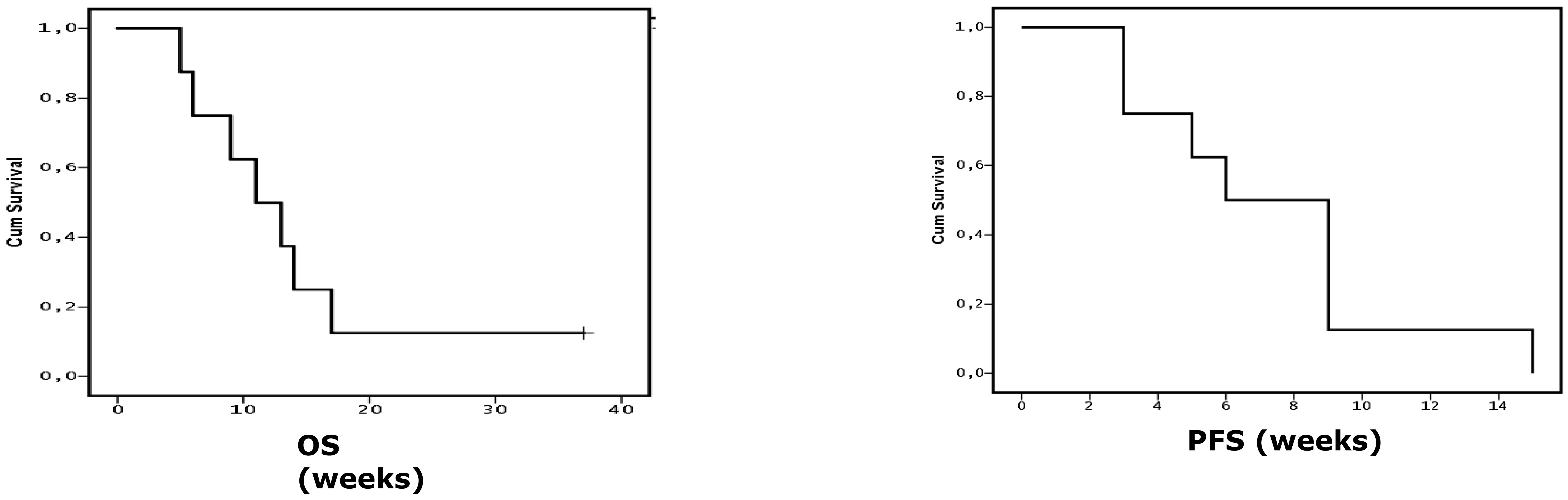

Supplement: Supplementary file 4 — Additional file 4: Figure S3. Kaplan–Meier survival curves for OS and PFS for patients with exclusively CNS PD and patients with BRAF/NRAS mutant copy number of > 500 copies/mL of plasma. (A) OS and PFS for the subgroup with PD exclusively in the CNS (n = 10), time is shown on the horizontal axis in weeks; (B) For Patient MEL36 the BRAFV600E/D mutation copy number is shown on the left vertical axis and the fractional abundance (% from the total cell-free DNA) on the right vertical axis. Time (in days) is shown on the horizontal axis. Illustrative CT or PET/CT images are shown on the upper part on the chart. The ctDNA was undetectable pretreatment with pembrolizumab when PD brain was observed on brain MRI and became detectable when the disease progressed as well in the visceral metastatic sites. (C) OS and PFS (in weeks) for the subgroup of patients with a baseline ctDNA copy number of > 500 copies/mL of plasma (n = 11) and (D) the subgroup of patients with a copy number of > 500 copies/mL of plasma at week 3 (n = 8). [file 12967_2019_2051_MOESM4_ESM.pdf]
